# Benzyl isothiocyanate alleviates neural damage in experimental autoimmune uveitis by modulating the TREM-1/PI3K/AKT signaling pathway

**DOI:** 10.3389/fphar.2026.1948992

**Published:** 2026-09-24

**Authors:** Sijie Liu, Wendan Yi, Yiqing Ji, Jingjing Zhang, Junli Zhang, Kejie Du, Xintong Zhao, Haowen Wang, Yichong Liu, Wenjuan Luo

**Affiliations:** 1 Department of Ophthalmology, The First Affiliated Hospital of Shenzhen University, Shenzhen Second People’s Hospital, Shenzhen, China; 2 Qingdao University Medical College, Qingdao, China

**Keywords:** benzyl isothiocyanate, experimental autoimmune uveitis, neuroprotection, oxidative stress, phagocytosis, TREM-1

## Abstract

**Purpose:** To investigate whether Benzyl isothiocyanate (BITC) can improve experimental autoimmune uveitis (EAU) and explore its potential mechanism. **Methods:** To evaluate the therapeutic efficacy of BITC, EAU was induced in C57BL/6 mice *via* immunization with IRBP_651-670_. Clinical scores and histopathological staining were employed to evaluate the severity of the disease. The integrity of the blood-retinal barrier (BRB) was assessed using Evans blue staining and Western blot analysis. Nissl staining, Brn3a immunofluorescence, and TUNEL assay were employed to assess retinal neuronal damage. *In vitro*, BV2 cells were exposed to lipopolysaccharide (LPS) and interferon-γ (IFN-γ). Biochemical markers of oxidative stress, mRNA levels of phagocytosis-related markers, protein and mRNA levels of neurotrophic factors, and triggering receptor expressed on myeloid cells-1 (TREM-1) were measured. Additionally, conditioned medium from BV2 cells was co-cultured with primary retinal ganglion cells (RGCs) to detect apoptosis. The functional relevance of TREM-1 in the protective effects of BITC was evaluated by overexpressing TREM-1 (oe-TREM-1). **Results:** In vivo, BITC alleviated clinical symptoms of EAU mice. Treatment significantly maintained BRB integrity and mitigated oxidative stress and neuronal damage. *In vitro*, BITC suppressed oxidative stress and phagocytic activity. The co-culture study of BV2 cells and primary RGCs showed that BITC can alleviate LPS + IFN-γ induced primary RGCs damage and inhibit apoptosis. Furthermore, BITC downregulated the expression of TREM-1/PI3K/AKT signaling pathway in EAU mice and LPS + IFN-γ induced BV2 cells. Oe-TREM-1 partially eliminated the inhibitory effects of BITC on oxidative stress and phagocytosis, as well as its protective effect against neuronal damage. **Conclusion:** BITC alleviates neural damage in EAU by down-regulating the TREM-1/PI3K/AKT signaling pathway, reducing oxidative stress and excessive phagocytosis, and promoting the secretion of neurotrophic factors.

## Graphical Abstract

**Figure:**
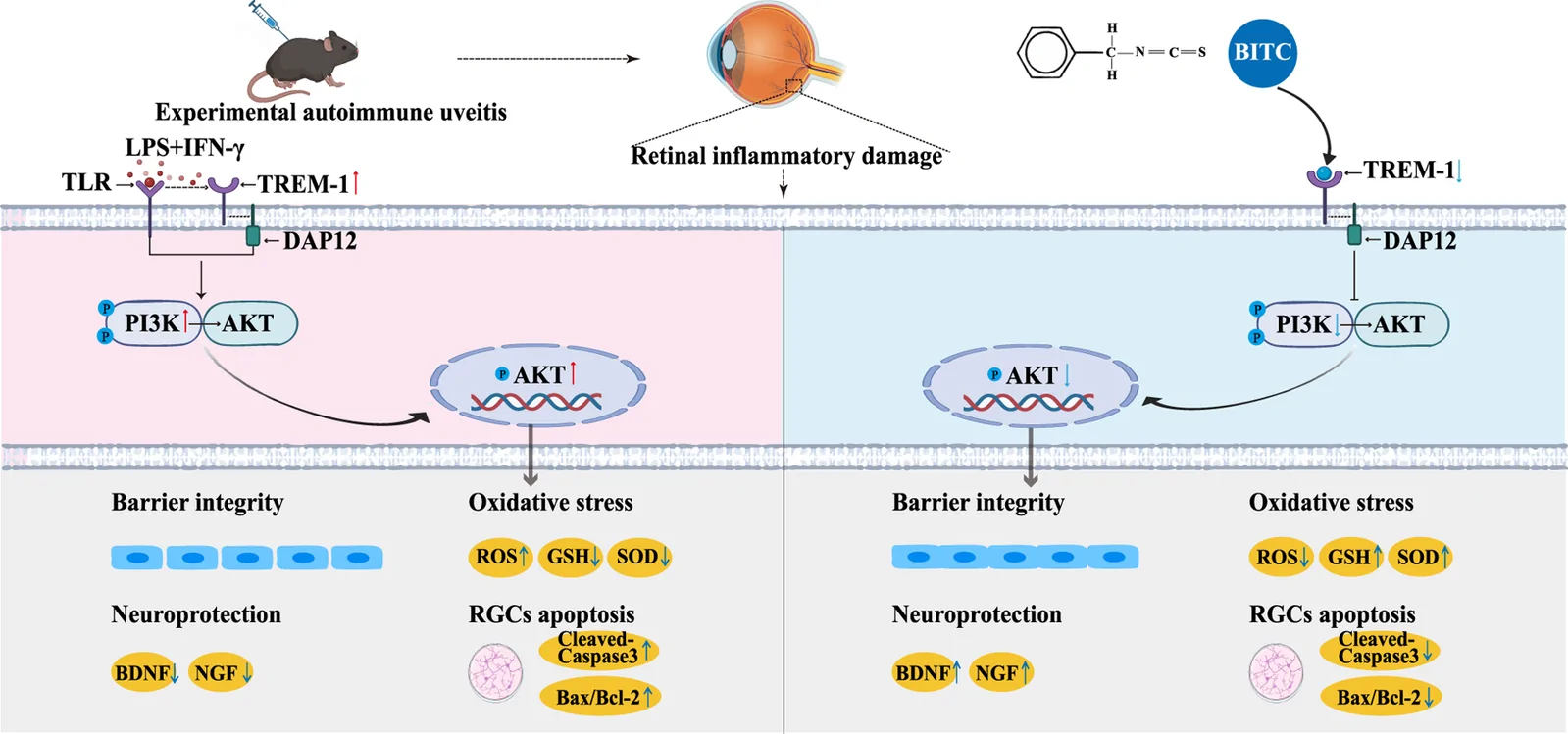

## 1 Introduction

Autoimmune uveitis (AU) constitutes a group of chronic, recurrent inflammatory diseases mediated by immune cells and inflammatory mediators, affecting intraocular tissues (Rosenbaum and Asquith, 2018; Hou et al., 2020; Yim et al., 2025; Guan et al., 2024). The experimental autoimmune uveitis (EAU) model is widely employed to study the pathogenic mechanisms and therapeutic responses of chronic uveitis (Shi et al., 2019). In EAU, activated immune cells interfere with the blood-retinal barrier (BRB), leading to retinal tissue damage and severe vision loss (Huang et al., 2018).

Research indicates that microglia primarily mediate the onset and progression of uveitis (Rao et al., 2003). Activated microglia release substantial amounts of reactive oxygen species (ROS) along with inflammatory cytokines, including TNF-α and IL-1β. These factors activate inflammatory signaling pathways mediated by nuclear factor κ-B (NF-κB), which further exacerbate BRB disruption and directly induce tissue oxidative damage (Chu et al., 2025; Chen J. et al., 2025; Flacco et al., 2026). Following retinal injury, microglia subsequently migrate into the outer nuclear layer (ONL) and sites of inflammation (Cao Y. et al., 2024), releasing neurotoxic factors such as ROS and hydrogen peroxide, while downregulating the synthesis and secretion of neurotrophic factors such as brain-derived neurotrophic factor (BDNF) and nerve growth factor (NGF). Changing the local microenvironment, weakening endogenous nutritional support for retinal ganglion cells (RGCs) (Gu et al., 2025; Khan et al., 2026), and inducing RGCs apoptosis (Shi et al., 2024; Qian et al., 2013). In the inflammatory microenvironment, excessively polarized pro-inflammatory microglia not only lose their ability to secrete BDNF and NGF, but also inhibit the expression of neurotrophic factors in other retinal cells such as Müller cells, *via* inflammatory mediators, thereby amplifying neuronal injury (Venanzi, McGee, and Hackam, 2025; Zou et al., 2026). Additionally, hyperactivated microglia may exhibit abnormal phagocytic behavior. Research indicates that excessive phagocytosis may disrupt functional synapses between RGCs and downstream neurons, leading to synaptic transmission disorders and impairing neural function (Wang Q. et al., 2025; Zhang D. Q. et al., 2025).

Triggering receptor expressed on myeloid cells-1 (TREM-1) is predominantly detected on microglia, monocytes/macrophages, and neutrophils (Bouchon et al., 2001; Peng et al., 2019). Increasing evidence indicates that injurious molecules such as lipopolysaccharide (LPS) trigger TREM-1 activation indirectly through ligation of Toll-like receptors (TLRs). Subsequently, TREM-1 binds to DNAX-activating protein 12 (DAP12) to activate downstream signaling cascades such as phosphatidylinositol 3-kinase/protein kinase B (PI3K/AKT). The synergistic action of TREM-1 and TLRs potentiates inflammatory reactions, inducing the marked release of pro-inflammatory cytokines and chemokines (Chen et al., 2024; Trivedi et al., 2025). Abnormal TREM-1 expression often drives excessive inflammatory damage, leading to tissue destruction and disease progression. Reports have shown that soluble TREM-1 (sTREM-1) levels are elevated in serum samples from patients with Behçet’s disease (BD) (Jung et al., 2011; Zhang et al., 2019), indicating that TREM-1 may contribute significantly to the pathogenesis during the active phase of autoimmune diseases. In studies of cerebral oedema, TREM-1 expression in brain tissue was markedly upregulated. TREM-1 inhibition improved neurological function and reduces neuronal apoptosis, while oeTREM-1 exacerbated injury (Chen J. et al., 2025).

Benzyl isothiocyanate (BITC) is a naturally bioactive compound derived from cruciferous plants, exhibiting significant anti-inflammatory and antioxidant activities (Yi et al., 2023; Lo et al., 2023; Liu et al., 2024). *In vitro* studies indicate that BITC modulates inflammatory markers production *via* the PI3K/Akt/NF-κB pathway, attenuating LPS-induced inflammation (Xiang et al., 2025). In diabetes research, BITC was found to ameliorate hyperglycemia induced oxidative stress, reduce cholesterol and triglyceride levels, and inhibit the increase in alanine aminotransferase and aspartate aminotransferase activities in type 2 diabetic mice, thereby improving hepatic function (Liu W. et al., 2025). However, it remains unknown whether BITC exerts a therapeutic effect against AU.

The main purpose of this study was to investigate the therapeutic potential of BITC in EAU. We employed an EAU mouse model, an LPS and interferon-γ (IFN-γ) induced BV2 cells inflammation model, and a BV2- primary RGCs co-culture system. This study found that the role of BITC is associated with the inhibition of the TREM-1/PI3K/AKT pathway, accompanied by the suppression of oxidative stress and microglial phagocytic function enhancement. These findings suggest that BITC may alleviate disease progression and neuroinflammation in EAU through the TREM-1 mediated signaling pathway.

## 2 Materials and methods

### 2.1 Animals

Male C57BL/6 mice (6–8 weeks of age) were acquired from Pengyue Company (Jinan, China). They were maintained in an air-conditioned room with a 12 h dark/light cycle at the animal research platform of the Affiliated Hospital of Qingdao University, and were given *ad libitum* access to chow and water. Mice were randomly divided into the various experimental groups. This study was conducted in accordance with the protocols approved by the Animal Research Ethics Committee of the Affiliated Hospital of Qingdao University. All experimental procedures were conducted in adherence to the Association for Research in Vision and Ophthalmology (ARVO) statement.

### 2.2 EAU induction and treatment

Human interphotoreceptor retinoid-binding protein (IRBP_651-670_, NovoProtein, Suzhou, China) was dissolved in phosphate buffered saline (PBS) at a concentration of 2 mg/mL, and emulsified with an equal volume of complete Freund’s adjuvant (Sigma, Missouri, United States) supplemented with 2.5 mg/mL *M. tuberculosis* strain H37Ra (Difco, New Jersey, United States). Using two syringes to repeatedly push through a three-way valve to form a water-in-oil emulsion. Mice were immunized subcutaneously with IRBP_651-670_ mixture in the nape, axilla, or inguinal regions. Simultaneously, pertussis toxin (List Labs, California, United States) was intraperitoneally injected at a concentration of 250 ng/mL per mouse, once on the day of immunization and once the following day, to enhance the success rate of the model (Shome et al., 2021; Shu et al., 2023). Early inflammation emerged 7–10 days post-immunization, with peak incidence observed between 14 and 21 days. After 7 days of EAU induction, all immunized mice underwent slit lamp examination to ensure successful modeling. On day 7 post-immunization, EAU mice were randomized into three groups using a computer-generated random number table. Daily intraperitoneal injections during the treatment period were administered by a technician who remained blinded to subsequent assessments. The first group of EAU mice received intraperitoneal injection of BITC (TargetMol, Massachusetts, United States) at 20 mg/kg/day, beginning on day 7 post-immunization (Sakaguchi et al., 2025). The second group received dexamethasone (DEX) as a positive control (1 mg/kg, 0.5 mL, IP) starting from day 7 after immunization (Yi et al., 2025). The third group received intraperitoneal administration of an equal volume of saline (200 μL per mouse). Unimmunized mice were employed as the control group (Liu X. et al., 2025). All mice with successful model establishment were included in the experiment. During this process, no mice were infected or died, and no mice were excluded. The subsequent EAU clinical scoring, retinal histological evaluation, and quantitative analysis of immunofluorescence images were all completed by two independent researchers who were unaware of the grouping.

### 2.3 Clinical scoring

The ocular inflammation in mice was photographed using a digital microscope (RWD, DOM-1001, Shenzhen, China). On the 14th day after EAU modeling, the clinical severity of ocular inflammation was evaluated by two independent workers who were not aware of the grouping, and scored 0–5 points based on five independent indicators, including corneal edema, conjunctival congestion, ciliary congestion, anterior chamber inflammatory infiltration, and posterior synechiae (Tian et al., 2011).

### 2.4 Evans blue (EB) staining

On day 14 after immunization, mice received 100 µL of 2% EB through the tail vein injection. Following circulation for 2 h, animals were sacrificed, their eyes were immediately enucleated and carried out in a 4% paraformaldehyde (Elabscience, Wuhan, China) for a duration of 2 h. Subsequently, all retinas were flat-mounted on glass slides, secured with coverslips, and prepared for observation under a fluorescence microscope (Nikon, Japan).

### 2.5 Hematoxylin and eosin (H&E) staining

Fourteen days after immunization, the eyeballs were collected and immersion-fixed in 4% paraformaldehyde buffer for 24 h. After paraffin embedding, tissues were cut along the papilla-optic nerve axis, sectioned at a thickness of 4 μm. And stained with H&E for examination under a light microscope (Nikon, Japan).

### 2.6 Nissl staining

Retinal tissue sections were subjected to Nissl staining to visualize the distribution of neuronal cell bodies within the retinal layers. The stained sections were analyzed under an optical microscope.

### 2.7 Retinal immunofluorescence staining

On the 14th day after immunization, the mouse eyeballs were removed and fixed. After peeling off the retina, permeate with 0.3% Triton X-100 and block with goat serum. The primary antibody ionized calcium-binding adapter molecule-1(Iba-1) (HuaBio, cat # ET1705-78, 1:200) was incubated overnight with the aforementioned retina. The next day, CoraLite ® Plus 488-goat-anti-rabbit recombinant secondary antibody (Proteintech, cat# RGAR002, 1:500) was applied and incubated for 1 h at room temperature in the dark (Table 1). 4′, 6-diamino-2-phenylindole (DAPI; Beyotime, Shanghai, China) was used for nuclear staining. Collect images using a 20x fluorescence microscope (Nikon, Japan). (Brn3a, red fluorescence, exposure time: 1s; DAPI, blue fluorescence, exposure time: 30 ms).

**TABLE 1:** Antibodies for western blot and immunofluorescence.

Antibody	Manufacturer	Cat. No	RRID
Anti-Brn3a	Abcam	EPR26313-54	AB317492
Anti-β-actin	Affinity Biosciences	AF7018	AB_2839420
Anti-ZO-1	Proteintech	21773-1-AP	AB_10733242
Anti-TREM-1	Proteintech	30496-1-AP	AB_3086337
Anti-IBa-1	HuaBio	ET1705-78	AB_3070606
Anti-Occludin	HuaBio	ET1701-76	AB_3070241
Anti-Bcl-2	HuaBio	ET1702-53	AB_3070315
Anti-Bax	HuaBio	ET1603-34	AB_3069679
Anti- Caspase-3	HuaBio	ER30804	AB_3069554
Anti-Cleaved Caspase-3	HuaBio	ET1602-47	AB_3069660
Anti-BDNF	HuaBio	ET1606-42	AB_3069737
Anti-NGF	HuaBio	ET1606-29	AB_3069730
Anti -p-PI3K	HuaBio	HA721672	AB_3072785
Anti - PI3K	HuaBio	ET1608-70	AB_3069823
Anti - p-AKT	HuaBio	ET1607-73	AB_2940863
Anti - AKT	HuaBio	ET1609-51	AB_2940862
488-Goat anti-rabbit secondary antibody	Proteintech	RGAR002	AB_3073506
594-Goat anti-rabbit secondary antibody	Proteintech	RGAR004	AB_3073508

Using paraffin-embedded retinal sections, deparaffinization with xylene, graded ethanol rehydration, rinse in distilled water, antigen retrieval, blocking, and permeabilization. Next, the samples were incubated overnight with Brn3a antibody (Bioss, cat#bs-3669R, 1:100) at 4 °C. Following primary incubation, they were treated with CoraLite® Plus 594-goat-anti-rabbit recombinant secondary antibody (Proteintech, cat#RGAR004, 1:500) for 1 h at room temperature in the dark (Table 1). We then counterstained the nuclei with DAPI (Beyotime). Fluorescence imaging was performed using a 20x fluorescence microscope to capture images. (Iba-1, green fluorescence, exposure time: 500 ms; DAPI, blue fluorescence, exposure time: 30 ms).

### 2.8 TUNEL staining

Apoptosis rates in retinal tissue and RGCs were detected using TUNEL staining according to the manufacturer’s protocol (Beyotime, Shanghai, China). Retinal sections were treated with DNase-free proteinase K at 37 °C for 30 min. RGCs were washed with PBS, fixed with 4% paraformaldehyde for 30 min, and permeabilized with 0.3% Triton X-100 for 10 min. Following washing, the TUNEL reaction mixture was prepared and incubated in the dark at 37 °C for 1 h. Subsequently, nuclei were counterstained using DAPI (Beyotime), followed by fluorescence imaging. Quantitative analysis of fluorescence intensity of apoptotic cells in the entire retina using ImageJ software. (Apoptotic cells, green fluorescence, exposure time: 400 ms; DAPI, blue fluorescence, exposure time: 30 ms).

### 2.9 Cell culture and treatment

BV2 cells (Procell, Wuhan, China) were maintained in DMEM with 10% FBS and 1% penicillin/streptomycin at 37 °C in a 5% CO_2_ atmosphere. At 80%–90% confluence, cells were seeded into 6-well plates at a density of 2 × 10^5^ cells/well and randomly assigned to three groups: Control, LPS (1 μg/mL) +IFN-γ (1 μg/mL), and LPS + IFN-γ + BITC (8 μM). 24 h post-seeding, BITC was added for 1 h pre-treatment. LPS + IFN-γ were then added, cells were harvested 24 h later.

### 2.10 Collection of conditioned medium

BV2 cells at 80% confluence were treated with LPS + IFN-γ and/or BITC for 6 h. After replacement, the cells were incubated for a further 24 h. The culture supernatants of BV2 cells were harvested and centrifuged at 1,000 rpm for 5 min to eliminate debris, yielding conditioned medium (CM). Groups comprised CM_Control_, CM_LPS+IFN-γ_, and CM_BITC_. If not used immediately, store the CM at −80 °C for subsequent experiments.

### 2.11 Enzyme-linked immunosorbent assay (ELISA)

On the 14th day of immunization in mice, the eyeballs were removed after euthanasia, and the retinal tissues were separated and placed in PBS for future use. BV2 cell culture supernatants were collected. The concentrations of IL-1β, IL-6, and TNF-α were measured using an ELISA kit (BioLegend, San Diego, CA, United States).

### 2.12 Isolation, purification of RGCs, and establishment of a co-culture system

Primary RGCs were isolated from 1-3-day-old C57 mice, which were kept in a standard breeding environment. Mice were euthanized *via* inhalation of isoflurane followed by cervical dislocation. Eyeballs were enucleated, and removed the surrounding muscles and connective tissues in cold PBS. Primary RGCs were purified following previously established protocols (Amina et al., 2021), with tissue incubation in isotonic buffer containing inorganic salts, glucose, papain, and DNase I for 30 min. The papain digestion was terminated using a low-ovomucoid solution. The retinal cell suspension was processed *via* a 5-min incubation with an anti-macrophage antibody (1:50, Fitzgerald Industries International, United States), followed by negative and positive selection using thymocyte differentiation antigen 1.2 antibody (1:50, Bio-Rad, United States) coated on culture plates (Choi et al., 2025). Poly-L-lysine solution (50 μg/mL) was precoated on plates for 2 h, wash thoroughly, and incubated overnight at 4 °C with Neurobasal medium containing laminin (10 μg/mL). Dried cells were treated with mouse laminin at 37 °C for 2 h. Primary RGCs were seeded onto laminin-coated plates in Neurobasal medium, with half of the medium replaced on day 3. To investigate the effects of activated BV2 cells on primary RGCs, primary RGCs were harvested as previously described and incubated in Neurobasal medium-coated wells overnight for attachment. CM was replaced. After an additional 24 h of incubation, and then proceed with subsequent experiments. In addition, to verify the direct neuroprotective effect of BITC on primary RGCs, BITC was added alone to the Neurobasal medium to treat primary RGCs, followed by the addition of LPS and IFN-γ. After a further 24 h of incubation, their survival rate was assessed.

### 2.13 Purity identification of primary RGCs

Using immunofluorescence detection technology to determine the purity of primary RGCs. The primary RGCs were fixed with 4% paraformaldehyde, permeabilized with 0.1% Triton X-100, and incubated at room temperature with a blocking solution containing 5% BSA for 1 h. Then incubated overnight with Brn3a antibody (Bioss, cat#bs-3669R, 1:100) at 4 °C. After washing, primary RGCs were incubated with goat anti-rabbit recombinant secondary antibody for 1 h. DAPI was used to label cell nuclei for fluorescence microscopy image acquisition. Primary RGCs were incubated with only the secondary antibody to serve as a negative control, thereby excluding non-specific staining induced by the secondary antibody. Using ImageJ software to count the total number of DAPI^+^ cells and BRN3A^+^ cells, primary RGC purity (%) = (number of Brn3a^+^ cells/total number of DAPI^+^ cells) × 100%. (Brn3a, green fluorescence, exposure time: 1s; DAPI, blue fluorescence, exposure time: 30 ms).

### 2.14 CCK-8 and lactic dehydrogenase (LDH) assays

Primary RGCs were seeded at 3,000 cells/well into 96-well plates. According to the instructions of the CCK-8 assay reagent (MCE, New Jersey, United States), measured the absorbance at 450 nm on a microplate reader.

LDH activity was quantified according to the LDH assay protocol (Beyotime). Quantify LDH release using a microplate reader at 490 nm.

### 2.15 Plasmid construction and cell transfection

The overexpression plasmid (trem1 mus) or control plasmid (pcDNA3.1+) was supplied by GenePharma (Shanghai, China), with verified sequence accuracy. Transfection was conducted according to the protocol for Lipofect5000 transfection reagent (BAIDAI, Changzhou, China), involving mixing Lipofect5000, transfection buffer, and overexpression plasmid in appropriate ratios. These mixtures were then added to wells at 80% confluency. Cells treated with an empty vector (oe-NC) served as controls, while cells transfected with the TREM-1 overexpression plasmid (oe-TREM-1) constituted the experimental group. Transfection efficiency was validated *via* real-time quantitative polymerase chain reaction (RT-qPCR) and Western blot analysis, followed by subsequent experiments. In addition, to verify the direct neuroprotective effect of BITC on RGCs, primary RGCs were treated with BITC alone in Neurobasal medium, followed by the addition of LPS and IFN - γ, and incubated for 24 h to detect their survival rate.

### 2.16 ROS assessment and flow cytometry

Quantitative analysis of intracellular ROS levels was performed using flow cytometry. Treated cells were gently washed twice with PBS, then incubated for 30 min at 37 °C in the dark with serum-free medium containing the 2′,7′-dichlorofluorescein diacetate (DCFH-DA) probe (Beyotime). Following incubation, cells were thoroughly washed twice with PBS to remove excess probe and resuspended in flow cytometry tubes. The cell suspension was immediately analyzed using a flow cytometer. The data were analyzed with FlowJo 10.9.0 software to determine the mean fluorescence intensity and visualize the results.

### 2.17 Detection of glutathione (GSH) and superoxide dismutase (SOD)

Mouse retinal tissue and BV2 cells were collected in pre-chilled PBS. GSH and SOD levels were measured using commercial assay kits.

### 2.18 Total RNA extraction and RT-qPCR

Total RNA was lysed and extracted according to the RNAeasy Reagent (Vazyme, Nanjing, China) protocol. The OneScript Hot 5X RT MasterMix (ABM, Jiangsu, China) was used for cDNA synthesis according to the manufacturer’s instructions. RT-qPCR was performed using qPCR mix reagent (Vazyme) to detect gene transcription product expression levels. Table 2 lists the sequences of all primers.

**TABLE 2:** Primers sequences for real-time qPCR.

Gene	Primer	Nucleotide sequences (5′-3′)
CD11b	Forward	ACG​CCA​TCT​ACA​TGA​TTG​TCA​C
​	Reverse	AAG​ACT​ACA​CTG​ACA​GGG​AGG​C
CD68	Forward	CTT​ATG​GAC​AGC​TTA​CCT​TT
​	Reverse	AGC​TCT​CGA​AGA​GAT​GAA​T
BDNF	Forward	TCT​ACG​AGA​CCA​AGT​GTA​ATC​C
​	Reverse	TTA​TGA​ACC​GCC​AGC​CAA​T
NGF	Forward	CGC​TCT​CCT​TCA​CAG​AGT​TTT
​	Reverse	GAC​ATT​ACG​CTA​TGC​ACC​TCA​GA
β-actin	Forward	ACG​GCC​AGG​TCA​TCA​CTA​TTG
​	Reverse	ACG​GCC​AGG​TCA​TCA​CTA​TTG

### 2.19 Western blot

Mouse retinal tissue, BV2 cells, and primary RGCs were lysed in RIPA buffer (Solarbio, Beijing, China) to extract total proteins. The BCA protein assay kit (Solarbio, Beijing, China) was used to measure protein concentrations. Western blotting was performed according to standard protocols, with blocking in 5% skimmed milk at room temperature for 2 h. Overnight incubation at 4 °C was conducted using the following primary antibodies: Zona Occludens-1 (ZO-1, 1:5,000), Occludin (1:2,000), B-cell lymphoma-2 (BCL-2, 1:2,000), BCL2-Associated X (Bax, 1:1,000), Cysteinyl Aspartate Specific Proteinase-3 (Caspase-3, 1:1,000), Cleaved Caspase-3 (1:1,000), TREM-1 (1:1,000), PI3K (1:2000), p-PI3K (1:1,000), AKT (1:1,000), p-AKT (1:1,000), Brain-Derived Neurotrophic Factor (BDNF, 1:10,000), Nerve Growth Factor (NGF, 1:2,000), β-actin (1:10,000). After overnight incubation, the membranes were incubated with horseradish peroxidase (HRP)-conjugated anti-rabbit IgG secondary antibodies (1:10,000) at room temperature for 1 h (Table 1). We detected protein bands with enhanced chemiluminescence (ECL, ZenBio, Chengdu, China), and their intensity values were analyzed using ImageJ software.

### 2.20 Phagocytosis assay

The phagocytic capacity of microglia was demonstrated by the uptake of fluorescent microspheres. BV2 cells were cultured and treated in 12-well plates. Each well was supplemented with a suspension of fluorescent latex microspheres (1μm, Sigma, United States). The cell plates were maintained at 37 °C for 2 h, followed by five washes with PBS (Cai et al., 2017). The sample was fixed in 4% paraformaldehyde, and stained it with DAPI. Imaging was performed using a fluorescence microscope.

### 2.21 Statistical analysis

Data analysis and bar chart generation were conducted using GraphPad Prism 9.0. Results are presented as mean ± standard deviation (SD). EAU clinical scores were analyzed using the Kruskal–Wallis test, with pairwise comparisons conducted *via* the Mann-Whitney U test. Statistical analysis involving three or more groups utilized one-way ANOVA, with *post hoc* pairwise comparisons performed using the Mann-Whitney U test. Comparisons between two groups were assessed using two-tailed Student's t-tests. All *in vivo* and *in vitro* experiments were based on a minimum of three independent biological replicates. A p-value <0.05 was considered statistically significant.

## 3 Results

### 3.1 BITC improved clinical signs in EAU mice

To investigate the effects of BITC on AU, EAU mice received BITC treatment concurrently with DEX as a positive control. On the 14th day after immunization, the anterior segment of the eye was dynamically observed using a digital microscope. The EAU group exhibited severe corneal oedema, ciliary hyperaemia, and inflammatory cell infiltration. Conversely, the BITC and DEX treated groups exhibited milder symptoms with significantly reduced clinical scores (Figures 1A–C). In H&E staining, the normal group showed an intact layered structure of the retina, while EAU mice exhibited significant retinal folds and disorganization of the photoreceptor cell layer. Both BITC and DEX therapy restored retinal morphology and markedly alleviated pathological damage (Figure 1D). Iba-1 immunostaining was employed to label activated microglia. Results indicated significantly increased Iba-1 positive staining intensity in the EAU group, indicating widespread activation of microglia. Activated Iba-1 positive cells exhibit a unique amoeboid morphology. After BITC or DEX treatment, morphological changes were inhibited and the fluorescence intensity of Iba-1 in the retina decreased. (Figure 1E). In summary, BITC prevented excessive activation of microglia and improved the prognosis of EAU mice.

**FIGURE 1:**
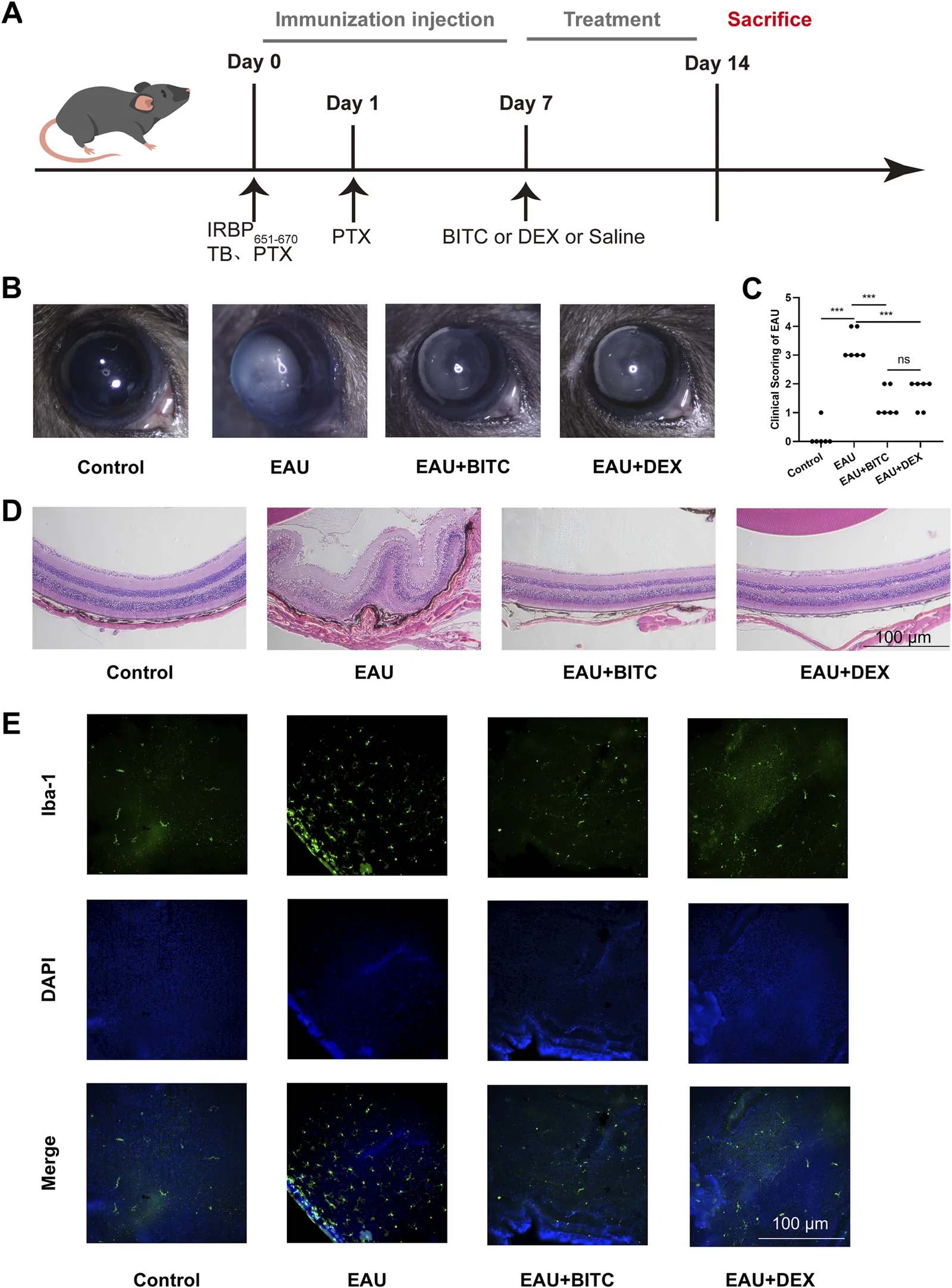
BITC improved the progression of EAU **(A)** Schematic diagram of EAU induction and treatment. **(B,C)** Micrographs of the anterior segment of mouse eyes, with clinical scores also shown (n = 6). **(D)** H&E-stained sections (scale bar: 100 μm). **(E)** Immunofluorescence staining images of microglial cells in retinal flat-mount (scale bar: 100 μm; Iba-1, green; DAPI, blue).

### 3.2 BITC ameliorated retinal inflammation, barrier disruption, and oxidative stress in EAU mice

To evaluate the effect of BITC on retinal inflammation, ELISA was used to detect the protein expression levels of IL-1β, IL-6, and TNF-α in the retinal tissues of mice in each group. As shown in Figures 2A–C, compared with the normal group, the expression of IL-1β, IL-6, and TNF-α in the retina of the EAU group was significantly increased. After BITC treatment, the levels of inflammatory factors in the EAU + BITC group were significantly lower than those in the EAU group. DEX as a positive control similarly reduced pro-inflammatory cytokine levels. BRB serves as a crucial protective barrier for the retina, shielding it from harmful substances in the bloodstream (Liu X. et al., 2025). The EB test was employed to assess the integrity of BRB. Contrary to the control, mice in the EAU demonstrated marked fluorescent leakage from retinal vessels, indicating severe BRB disruption. Conversely, the BITC and DEX-treated groups demonstrated reduced vascular leakage and diminished staining of surrounding tissues (Figure 2D). As shown by Western blot, the expression levels of tight junction proteins ZO-1 and Occludin were decreased in EAU mouse retina. BITC upregulated the expression of ZO-1 and Occludin in the retina of EAU mice. The observed trend of its effects was consistent with that of the DEX group. (Figures 2E–G). Further assessment of oxidative stress levels in EAU mouse retina reveals a significant reduction in total GSH content and SOD activity compared to controls. Treatment with BITC or DEX markedly restores GSH and SOD levels, approaching those of the control group, indicating that BITC effectively enhance retinal antioxidant capacity and partially counteract EAU-induced oxidative damage (Figures 2H,I). In summary, BITC demonstrated the ability to protect the BRB and exerts antioxidant effects.

**FIGURE 2:**
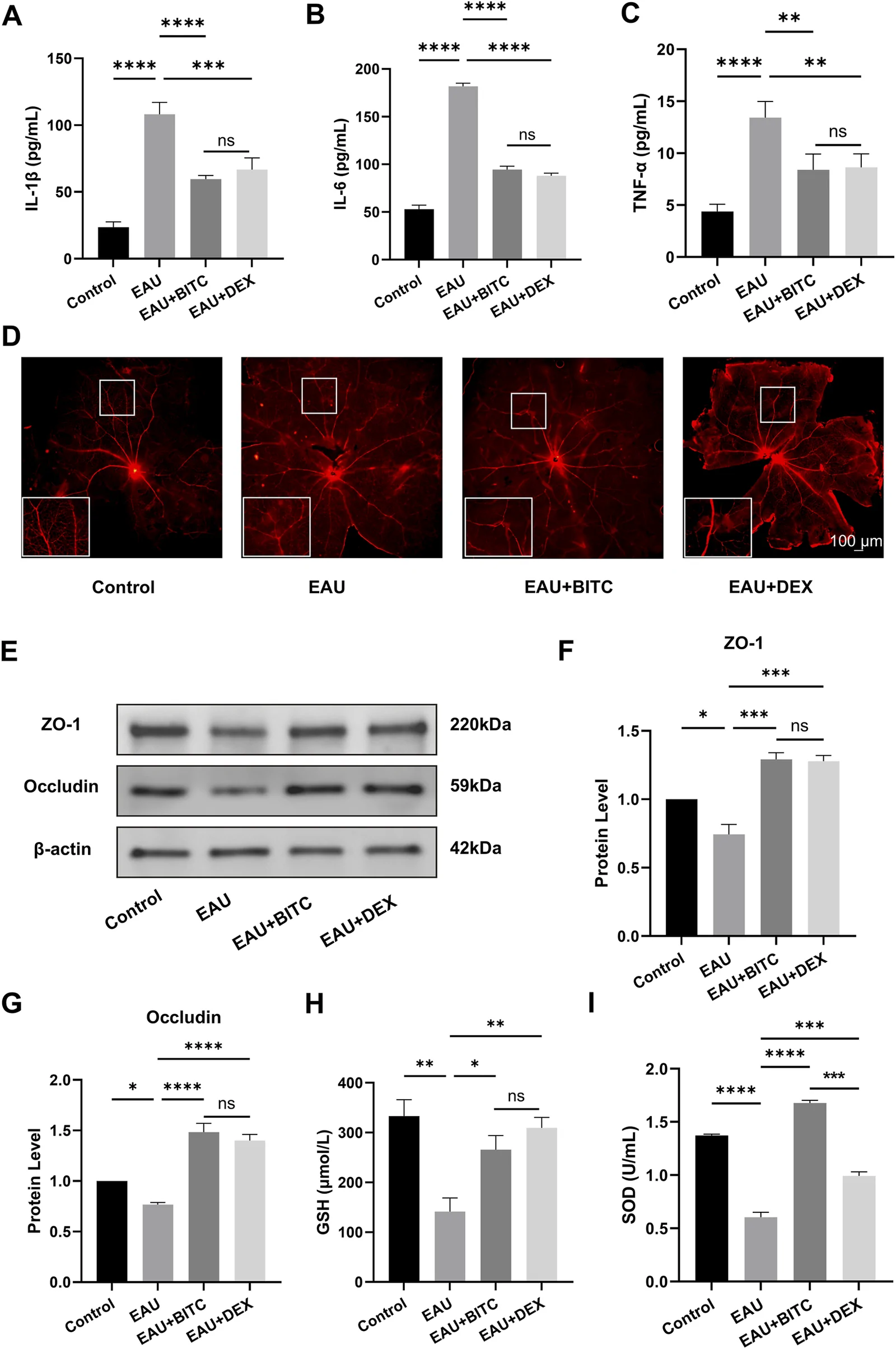
BITC ameliorated retinal inflammation, barrier disruption, and oxidative stress in EAU mice. **(A–C)** Proinflammatory cytokine protein levels in mouse retina (*n* = 3). **(D)** EB staining of mouse retinal flat mounts (scale bar: 100 μm). **(E–G)** Quantitative analysis of ZO-1 and Occludin protein levels in mouse retinal tissue. **(H,I)** The levels of GSH **(H)** and SOD **(I)** in mouse retinal tissue. Bar charts are presented as mean ± SD. *P < 0.05, **P < 0.01, ***P < 0.001, and ****P < 0.0001 denote significant differences compared with the EAU group.

### 3.3 BITC mitigates retinal neuron damage and apoptosis in EAU mice

To determine the impact of BITC on the retina, we first employed TUNEL staining to assess apoptosis in retinal full-thickness cells (Figures 3A,B). The control group exhibited only a minimal number of TUNEL-positive cells. The EAU group showed extensive TUNEL positive cells in the entire retina, particularly within the inner nuclear layer (INL) and ganglion cell layer (GCL), indicating that the primary damage occurred within the retinal neural layers. BITC treatment markedly attenuated the fluorescence intensity of INL and GCL layers. Subsequently, we employed Nissl staining to observe the state of retinal neurons. As depicted in Figure 3C, compared to the control group, all retinal cells in the EAU group exhibited reduced staining intensity, particularly within the GCL layers. Neurons in these layers displayed significant edema, accompanied by a marked reduction in the number of Nissl bodies, which were sparsely and irregularly distributed. In contrast to the EAU group, BITC treatment partially restored neuronal density and Nissl body distribution, with cells exhibiting relatively regular arrangement. Furthermore, we directly assessed the quantity and survival of RGCs through staining with the RGCs specific marker Brn3a. We observed tightly arranged Brn3a-positive cells in the GCL layer of normal mice. Following EAU induction, the number of Brn3a-positive cells in the retina decreased significantly, while the BITC group exhibited enhanced fluorescence intensity in the GCL layer (Figures 3D,E). On the molecular level, Western blot was employed to assess the protein expression of BDNF and NGF (Figures 3F–H). Compared to the control group, the EAU mouse retina showed a marked downregulation of BDNF and NGF proteins. BITC administration significantly upregulated the expression of both neurotrophic factors. In summary, BITC treatment effectively mitigated retinal neuronal damage in the EAU model.

**FIGURE 3:**
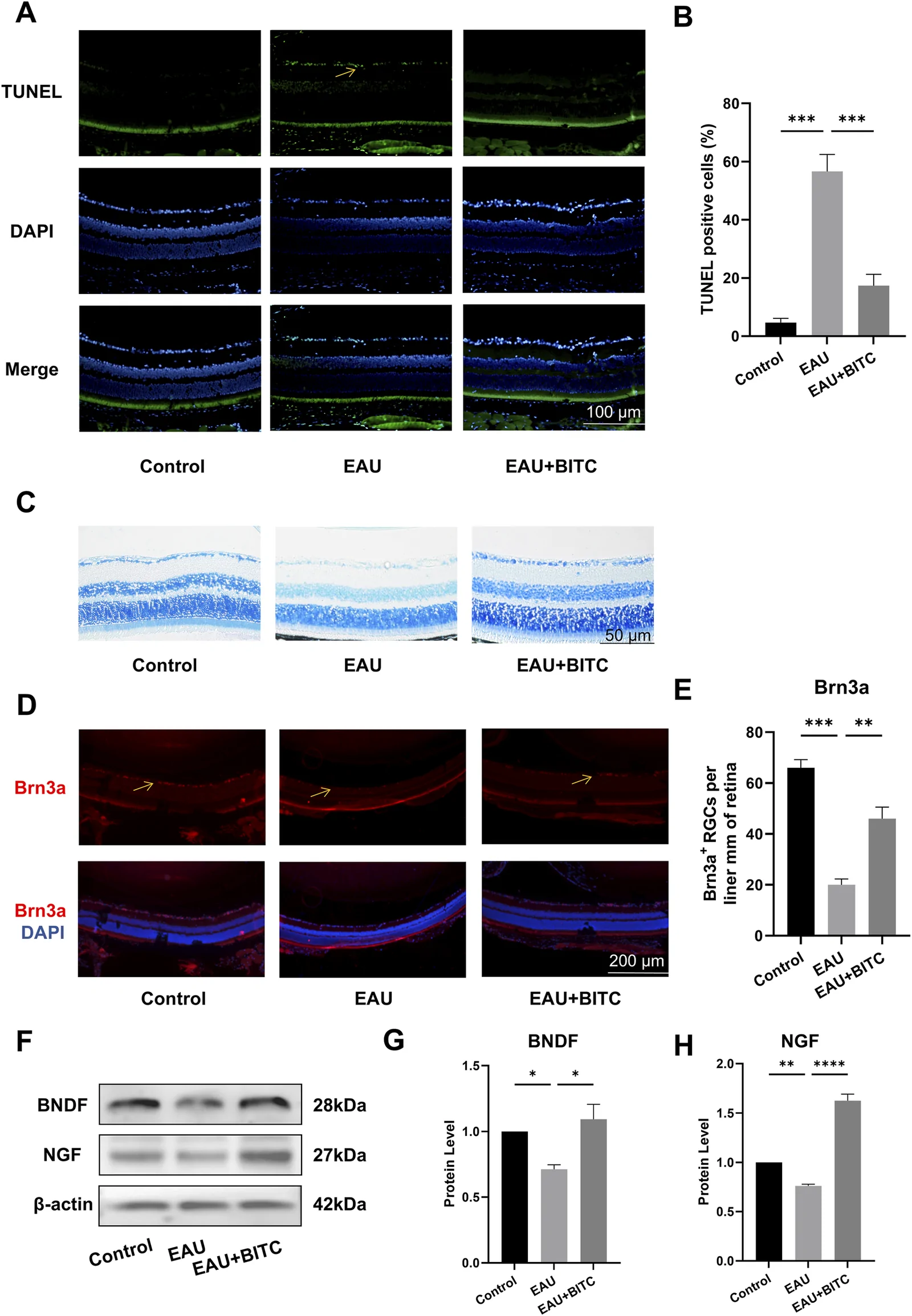
BITC exerted neuroprotective effects in EAU mice. **(A,B)** TUNEL assay of mouse retina sections (scale bar: 100 μm, yellow arrow indicates TUNEL^+^ cells; DAPI, blue). **(C)** Nissl staining images (scale bar: 50 μm). **(D)** Brn3a immunofluorescence staining of mouse retina sections (scale bar: 200 μm, yellow arrow indicates Brn3a^+^ cells). **(E)** Quantitative analysis of retinal RGCs expression. **(F–H)** Protein expression levels of BDNF and NGF in mouse retina and their related statistical analysis. Bar graphs represent mean ± SD. *P < 0.05, **P < 0.01, ***P < 0.001, and ****P < 0.0001 denote significant differences compared with the EAU group.

### 3.4 BITC inhibited inflammation, oxidative stress and phagocytic activity of BV2 cells

By stimulating BV2 cells with LPS + IFN-γ, the pathogenesis and treatment strategies of uveitis can be studied at the cellular level. Our team previously conducted drug toxicity testing *via* the CCK8 assay (Yi et al., 2026), identifying a safe concentration of BITC acting on BV2 cells at 8 μM. Detect the protein levels of IL-1 β, IL-6, and TNF-α in the supernatant of BV2 cells using ELISA. As shown in Figures 4A–C, compared with the control group, the expression of three inflammatory factors was significantly increased in the LPS + IFN - γ group. After treatment with BITC, the levels of IL-1β, IL-6, and TNF-α in the supernatant of the LPS + IFN-γ+BITC group were significantly reduced compared to the LPS + IFN-γ group. Using flow cytometry to assess ROS production, we observed increased ROS generation in the LPS + IFN-γ group, with a significant reduction in the BITC treatment group (Figures 4D,E). Similarly, levels of GSH and SOD decreased in the LPS + IFN-γ group, but these reductions were reversed following BITC administration (Figures 4F,G). Furthermore, we investigated BITC’s effects on phagocytosis. The phagocytic activity of BV2 cells was markedly enhanced in the LPS + IFN-γ group compared to control, manifested as an accumulation of 7.7 ± 0.9 microbeads per cell. BITC treatment reduced this to an average of 3.5 ± 0.3 beads per cell (Figures 4J,K). The mRNA expression levels of phagocytic markers CD11b and CD68 were significantly increased in the LPS + IFN-γ group compared to the control, and were markedly reduced following BITC treatment (Figures 4H,I). Overall, BITC alleviated LPS + IFN-γ induced inflammation, oxidative stress, and enhanced phagocytic activity in BV2 cells.

**FIGURE 4:**
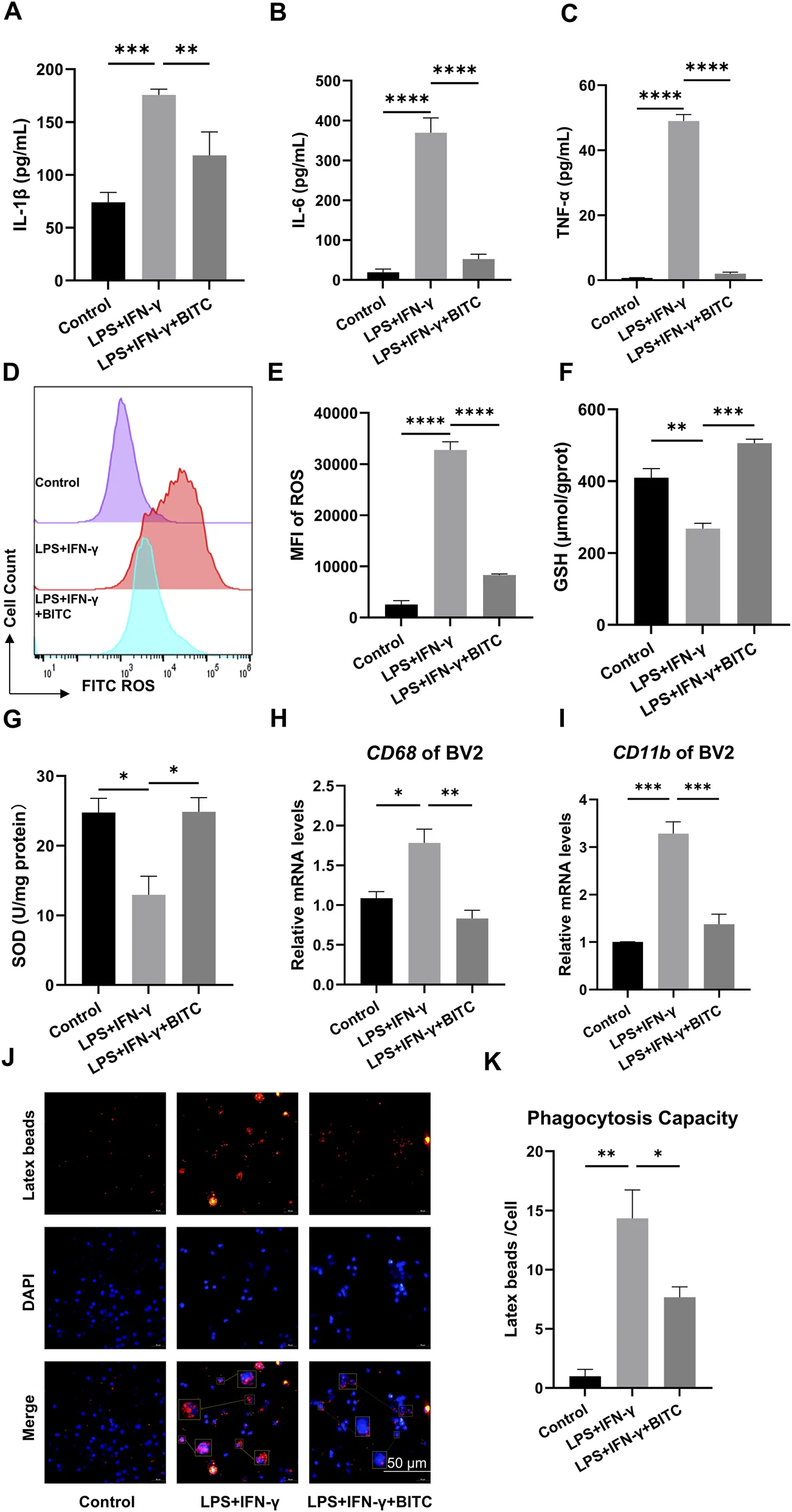
BITC inhibited inflammation, oxidative stress and phagocytic activity of microglia. **(A–C)** Proinflammatory cytokine protein levels in BV2 cells (*n* = 3). **(D,E)** Flow cytometry analysis of intracellular ROS levels, presented as mean fluorescence intensity (MFI). **(F,G)** The intracellular levels of GSH (F) and SOD (G) in BV2 cells. **(H,I)** RT-qPCR analysis of CD68(H) and CD11b(I) mRNA expression. **(J,K)** The effect of BITC on the excessive phagocytosis of BV2 cells induced by LPS + IFN-γ, including quantitative analysis. Bar charts are presented as mean ± SD. *P < 0.05, **P < 0.01, ***P < 0.001, and ****P < 0.0001 denote significant differences compared with the LPS + IFN-γ group.

### 3.5 BITC can reduce apoptosis of primary RGCs *in vitro*

We evaluated the effect of BITC on neurotrophic factor secretion in BV2 cells. As shown in Figure 5A, following LPS + IFN-γ stimulation, the protein expression levels of BDNF and NGF were significantly decreased. Treatment with BITC effectively reversed the inhibitory effects of LPS + IFN-γ, markedly upregulating the expression of BDNF and NGF (Figures 5B,C). Similarly, mRNA levels of BDNF and NGF were downregulated after LPS + IFN-γ stimulation and increased following BITC treatment (Supplementary Figure S1A,B). The purity testing of primary RGCs can be found in Figure 5D. The negative control image of only secondary antibody staining can be found in Supplementary Figure S3. We collected CMs from different treatments for processing primary RGC cells (Figure 5E). CCK-8 assays revealed that the viability of primary RGCs was markedly reduced in CM_LPS+IFN-γ_ conditions. However, CM_BITC_ treatment significantly enhanced the survival rates of primary RGCs (Figure 5F). Similarly, LDH release was increased in the CM_LPS+IFN-γ_ group, but decreased after CM_BITC_ treatment, indicating reduced cell death (Figure 5G). The above results suggest that BITC can indirectly exert a protective effect on primary RGCs by regulating the function of BV2 cells. Furthermore, we verified whether BITC has a direct neuroprotective effect on primary RGCs. The results showed that compared with the LPS + IFN-γ group, the BITC pretreatment group did not significantly improve the survival rate of primary RGCs (Supplementary Figure S2), indicating that BITC lacks a direct neuroprotective effect on primary RGCs.

**FIGURE 5:**
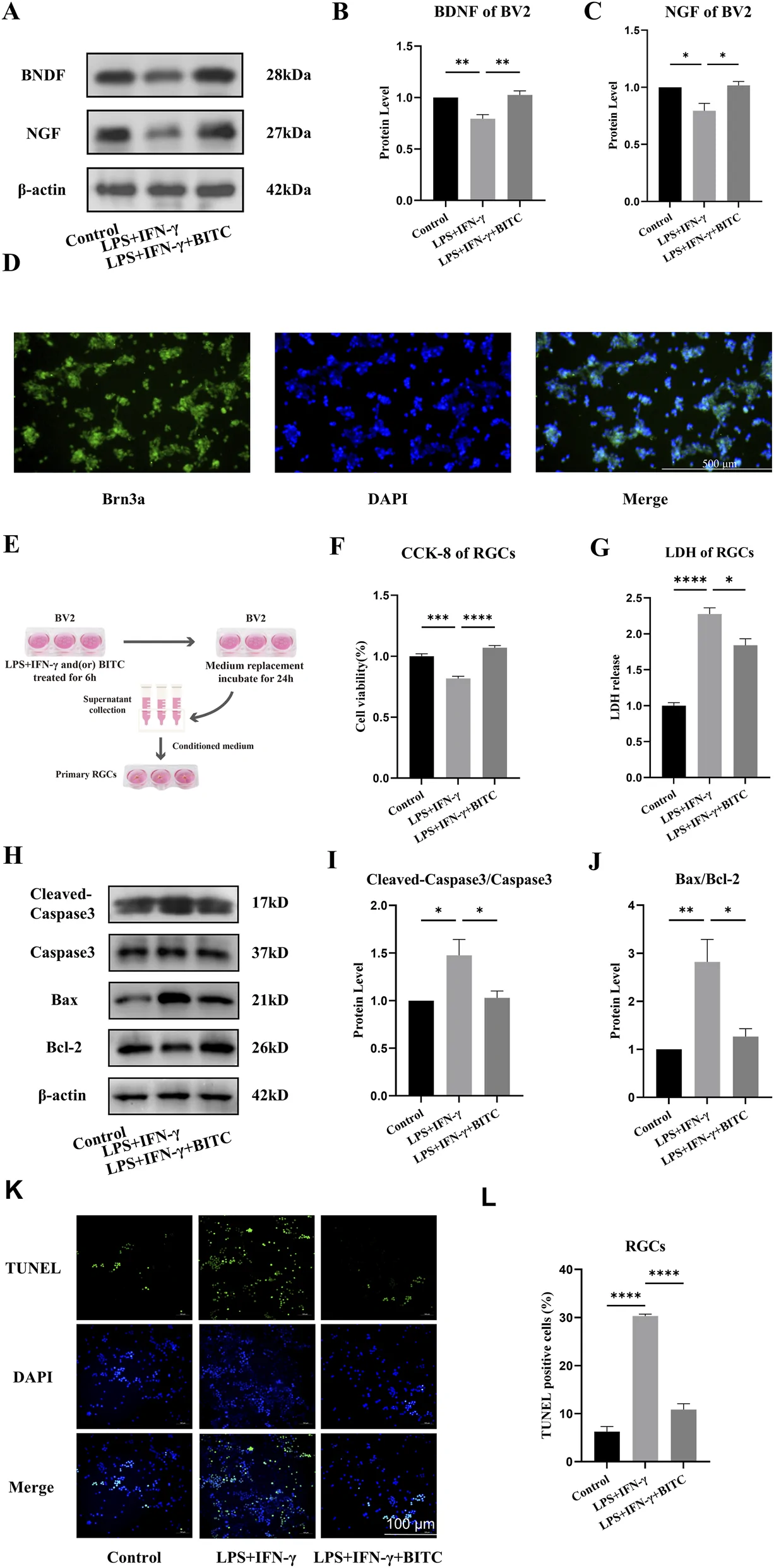
BITC exerted neuroprotective effects *in vitro*. **(A–C)** Protein expression levels of BDNF and NGF in BV2 cells and associated statistical analysis. **(D)** Purity detection of primary RGCs (green fluorescence: Brn3a^+^ cells, blue fluorescence: DAPI, scale bar: 200 μm, Purity of primary RGC: 88.69% ± 0.7864%, n = 3). **(E)** Schematic diagram of BV2-primary RGCs co-culture experiment. **(F)** Survival ability of primary RGCs after different CM treatments. **(G)** LDH production in primary RGCs after different CM treatments. **(H,J)** Protein levels of Cleaved-Caspase3/Caspase3 and Bax/Bcl-2 in primary RGCs and corresponding statistics. **(K,L)** TUNEL assay of primary RGCs (scale bar: 100 μm). Bar graphs represent the mean ± SD. *P < 0.05, **P < 0.01, ***P < 0.001, and ****P < 0.0001 denote significant differences compared with the CM_LPS+IFN-γ_ group.

Apoptosis is a common form of RGCs death (Huang et al., 2026). Western blot results indicated that CM_LPS+IFN-γ_ stimulation increased the expression of Cleaved-Caspase3 and Bax/Bcl-2 ratio. BITC significantly decreased the levels of Cleaved-Caspase3 and Bax (Figures 5H–J), suggesting its role in neuroprotection through modulating apoptotic pathways. The effect of CM on primary RGC apoptosis was directly examined by TUNEL staining (Figure 5K). Primary RGCs cultured with CM_LPS+IFN-γ_ showed an elevated apoptotic rate compared to the control, which decreased upon BITC treatment (Figure 5L). In summary, BITC promoted the secretion of neurotrophic factors by BV2 cells, indirectly alleviating the apoptosis of primary RGCs.

### 3.6 BITC regulated the TREM-1/PI3K/AKT signaling pathway

Research indicates that TREM-1 is critically involved in oxidative stress and neural injury (Al Sawy et al., 2025; Shen et al., 2024). Consequently, we observed that TREM-1 protein levels were notably increased in the retinal tissue of EAU mice relative to control. Following BITC treatment, TREM-1 expression was effectively suppressed (Figure 6A). Similarly, *in vitro*, stimulation of BV2 cells with LPS + IFN-γ markedly upregulated TREM-1 protein and mRNA expression, which was reversed by BITC treatment (Figures 6B,C). Further investigation into the signaling pathways modulated by BITC, as depicted in Figure 6D, revealed hyperactivation of the PI3K/AKT phosphorylation in the retinas of EAU mice, which was effectively suppressed by BITC therapy (Figures 6E,F). *In vitro*, BITC inhibits the levels of p-PI3K and p-AKT stimulated by LPS + IFN-γ (Figures 6G–I). In conclusion, the experiments demonstrate that BITC inhibits the hyperactivation of the TREM-1 and PI3K/AKT signaling pathways.

**FIGURE 6:**
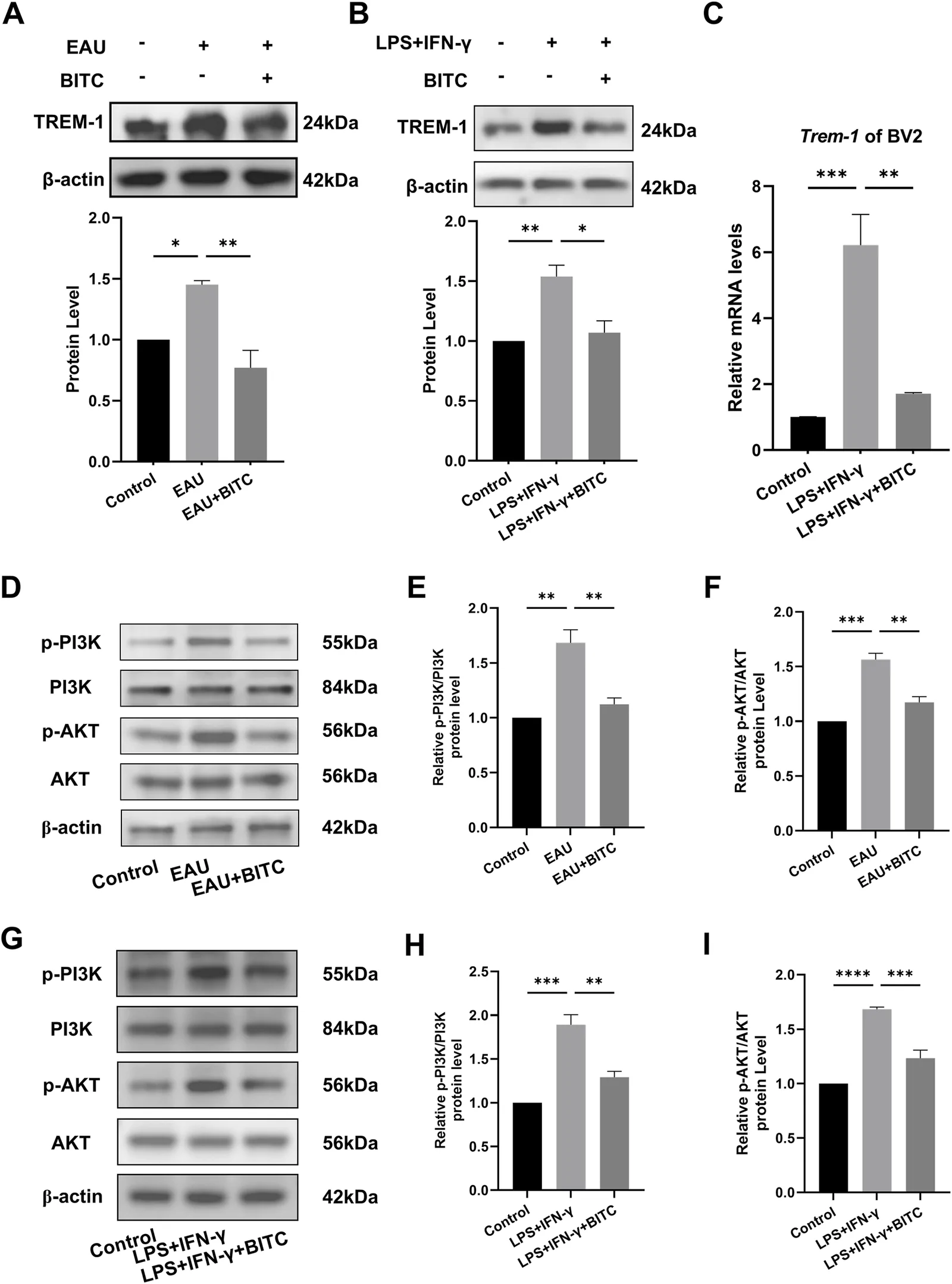
BITC regulated the TREM-1/PI3K/AKT signaling pathway. **(A,B)** Protein expression and statistical data of TREM-1 in mouse retina **(A)** and BV2 cells **(B)**. **(C)** mRNA level of TREM-1 in BV2 cells. **(D–I)** Western blot analysis and quantification of PI3K/AKT pathway protein expression levels in mouse retina **(D–F)** and BV2 cells **(G–I)**. Bar graphs represent the mean ± SD. *P < 0.05, **P < 0.01, ***P < 0.001, and ****P < 0.0001 denote significant differences compared with the EAU or LPS + IFN-γ group.

### 3.7 TREM-1 is a functionally relevant mediator of the protective effect of BITC

To clarify whether BITC exerts therapeutic effects by modulating TREM-1. We transfected BV2 cells with oe-NC or oe-TREM-1. The mRNA levels of oe-TREM-1 were upregulated nearly two thousand-fold, while protein levels increased approximately two-fold, confirming successful establishment of the overexpression model (Figures 7A–C). Western blot analysis revealed that oe-TREM-1 further activated the phosphorylation of the PI3K/AKT pathway. In the overexpression model, the inhibitory effect of BITC on the phosphorylation pathway was significantly weakened (Figures 7D–F). Regarding oxidative stress, it was observed in the oe-TREM-1 model that the levels of GSH and SOD further decreased in the LPS + IFN-γ group, concomitant with a diminished capacity of BITC to elevate these antioxidants (Figures 7G,H). In terms of phagocytosis, compared to the oe-NC + LPS + IFN-γ group, the oe-TREM-1+LPS + IFN-γ group exhibited significantly elevated CD68 and CD11b expression, further enhancing phagocytic activation. When TREM-1 was overexpressed, the inhibitory effect of BITC on phagocytic uptake is partially counteracted (Figures 7I,J). Additionally, we assessed the expression of BDNF and NGF. Oe-TREM-1 further enhanced the LPS + IFN-γ induced downregulation of these neurotrophins, partially blocking BITC’s upregulation effect on BDNF and NGF (Figures 7K–M). Based on the above findings, the role of BITC in reducing oxidative stress and phagocytosis, and promoting the secretion of neurotrophic factors may be related to its inhibition of TREM-1/PI3K/AKT.

**FIGURE 7:**
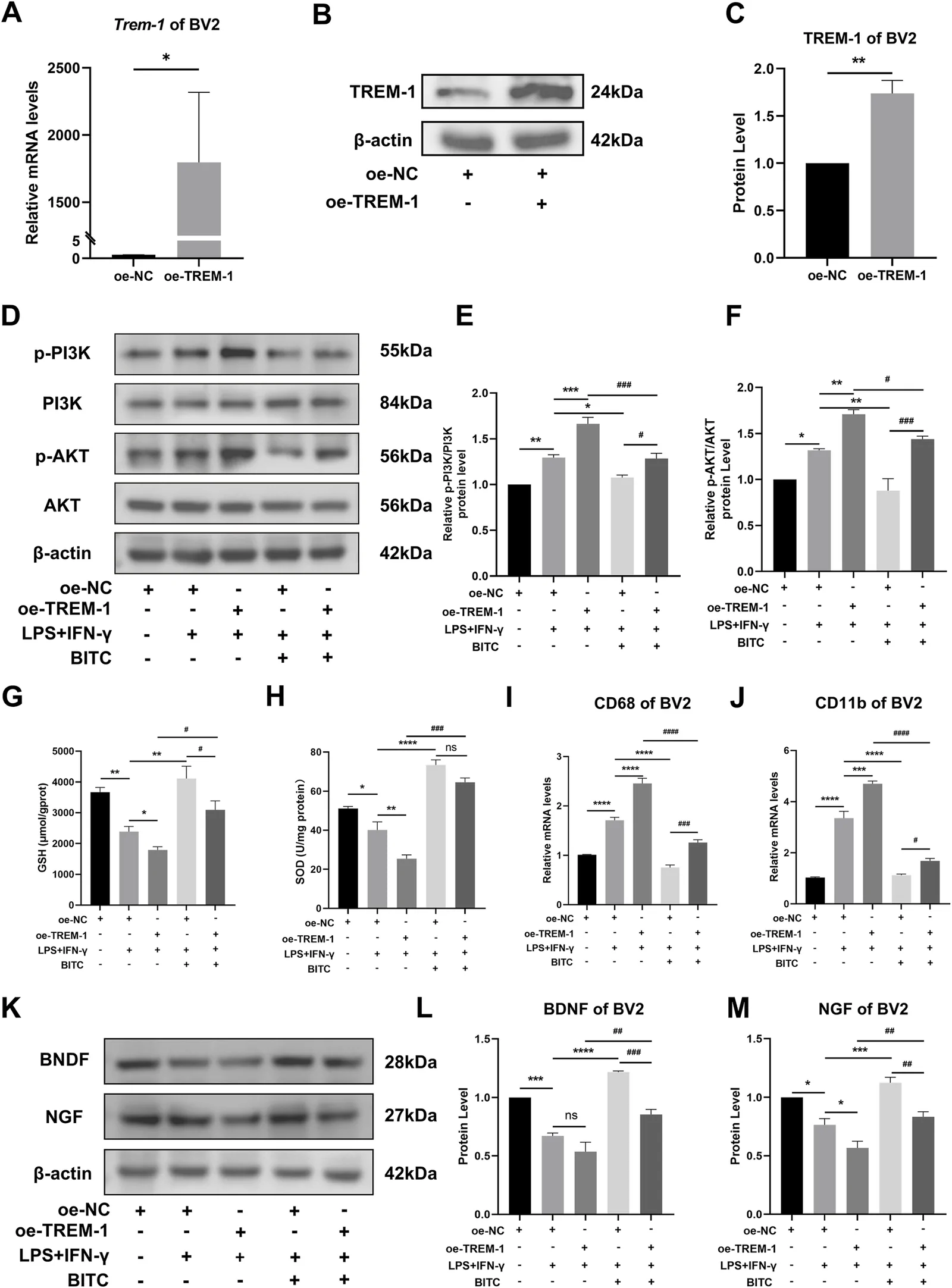
TREM-1 is a functionally relevant mediator of the protective effect of BITC **(A)** mRNA levels of TREM-1 in BV2 cells. **(B,C)** Western blot images of TREM-1 in BV2 cells. **(D–F)** Expression levels of proteins in the PI3K/AKT pathway in BV2 cells. **(G,H)** Contents of GSH **(G)** and SOD **(H)** in BV2 cells. **(I,J)** mRNA levels of CD68 **(I)** and CD11b **(J)** in BV2 cells. **(K–M)** Western blot images of BDNF and NGF in BV2 cells. Bar graphs represent the mean ± SD. *P < 0.05, **P < 0.01, ***P < 0.001, and ****P < 0.0001 denote significant differences compared with the oe-NC + LPS + IFN-γ. #P < 0.05, ##P < 0.01, ###P < 0.001, and ####P < 0.0001 denote significant differences compared with the oe-TREM-1+LPS + IFN-γ + BITCgroup.

## 4 Discussion

Our findings provided evidence that BITC could mitigate the progression of uveitis. Specifically, BITC treatment is associated with the protection of BRB integrity, alleviation of retinal oxidative stress damage, and inhibition of microglial oxidative stress and phagocytic function enhancement. We further found that BITC can alleviate retinal neuronal injury in EAU mice. Based on this discovery, we designed an indirect co culture experiment between BV2 cells and primary RGCs. To investigate the apoptotic injury of primary RGCs, they were co-cultured with CM. At the molecular mechanism level, BITC treatment was associated with TREM-1 downregulation and PI3K/AKT signaling suppression. The partial reversal of the protective effect of BITC by oe-TREM-1 further confirms that TREM-1 may be involved in regulating the PI3K/AKT signaling pathway and the protective effect of BITC. These findings suggest that BITC may be a potential treatment for EAU.

IRBP_651-670_ induced EAU triggers persistent inflammatory infiltration within mouse retinal tissue, accompanied by disruption of the BRB integrity and significant oxidative stress damage (Cao F. et al., 2024). Our findings indicated that severe retinal vascular leakage in EAU mice, significant downregulation of tight junction protein expression, and increased secretion of inflammatory factors. There is a cascade amplification effect between retinal inflammation and BRB injury in the EAU process. Inflammatory infiltration can further exacerbate retinal barrier leakage, while BRB integrity damage can promote the continuous invasion of peripheral inflammatory cells and inflammatory mediators into retinal tissue, forming a malignant cycle of sustained inflammation activation. Research by Chen H et al. (2025) confirmed that excessive ROS leads to decreased expression of ZO-1 and Occludin proteins, compromising BRB integrity and exacerbating retinal injury (Chen J. et al., 2025). Following BITC treatment, the secretion of inflammatory factors decreases, BRB leakage diminished, and the levels of ZO-1 and Occludin within the retina were stably upregulated, thereby maintaining barrier function. BRB disruption not only leads to plasma component extravasation, resulting in retinal oedema (Fan and Li, 2025). More significantly, it disturbs the intrinsic homeostasis of the retina, severely impairing its antioxidant defense system. In EAU mice, we observed decreased GSH and SOD, indicating a shift in redox balance towards oxidative stress. As reported by El Badawy SA et al. (2021) in a rat model of gastric injury, BITC modulated oxidative stress *via* the nuclear factor E2-related factor 2 (Nrf2)/heme oxygenase-1 (HO-1) and NF-κB signaling pathways, notably increasing GSH and SOD levels, demonstrating potent antioxidant effects (El Badawy et al., 2021). Similarly, in our study, BITC treatment significantly elevated antioxidant levels. Therefore, we hypothesize that the protective effects of BITC partly derive from enhancing the antioxidant defense capacity of retinal tissue and BV2 cells, thereby mitigating oxidative damage induced by inflammation.

Microglia constitute the key innate immune cells within the eye, serving as sentinels and regulators of retinal homeostasis. They continuously participate in immune surveillance of the surrounding neural tissues, which modulate inflammation related to infection and autoimmunity (Feng et al., 2024). Resting microglia exhibit highly branched processes (Silverman and Wong, 2018). Upon activation, microglia rapidly retract their fine projections, migrate to the injured site, generate inflammatory mediators, and exert phagocytic functions to clear invading microorganisms and cellular debris. This timely and regulated phagocytic activity is fundamental to the innate immune defense of the central nervous system. However, under persistent and severe abnormal stimulation, microglial phagocytic function may become dysregulated. Rather than being confined to clearing harmful substances, they may mistakenly attack healthy neuronal tissue (Fan et al., 2022). In neuropsychiatric research, excessive microglial activation and aberrant synaptic phagocytosis have been implicated in the progression of depression. Acupuncture treatment has been shown to inhibit such abnormal phagocytosis, significantly reducing neuronal damage and alleviating depressive symptoms (Zhang et al., 2026). The phagocytic function of microglia is the core of its role in maintaining homeostasis and immune defense in the nervous system, and CD11b and CD68 are two key molecules that characterize this functional state (Rivi et al., 2025). CD11b is an important phagocytic receptor on the surface of microglia, mediating their adhesion, internalization, and phagocytosis of targets (Cruz-Cárdenas et al., 2026). CD68 is a transmembrane glycoprotein primarily localized in lysosomes and widely used as a histological indicator of microglial phagocytic activity (Wang et al., 2026). In the obesity model, CD11b and CD68 double positive cells can encapsulate neurons, indicating the presence of enhanced synaptic phagocytic receptors (Rong et al., 2025). The phagocytosis of microglia plays a dual role in retinal homeostasis. On the one hand, physiological phagocytic behavior is a prerequisite for maintaining retinal homeostasis. On the other hand, excessive abnormal phagocytosis of microglia can directly damage the integrity of BRB. It is reported that in diabetes retinopathy, abnormal phagocytosis of microglia will intensify vascular leakage, leading to the destruction of BRB integrity and new capillary formation. Stimulation with LPS and IFN-γ induces sustained microglial activation (Zhang H. et al., 2025). In the present study, LPS and IFN-γ stimulated BV2 cells exhibited enhanced phagocytic function, accompanied by a significant upregulation of phagocytic markers CD68 and CD11b at the mRNA level, which was consistent with previous research on the enhanced phagocytic function of BV2 cells under oxidative stress and inflammatory stimulation (Rivi et al., 2025). BITC treatment markedly suppressed the excessive phagocytosis induced by LPS and IFN-γ, along with decreased mRNA expression of CD11b and CD68, suggesting that BITC may exert protective effects by inhibiting hyperactive microglial phagocytosis.

RGCs are pivotal neurons in visual signal transmission and play a critical role in relaying visual information from the retina to higher visual centers (Choi et al., 2025). Dysfunction or death of RGCs directly results in interruption of optic nerve signal conduction, clinically manifesting as irreversible visual impairment (Huang et al., 2026). In the EAU model, TUNEL and neuronal staining confirmed retinal neuronal apoptosis and a reduction in RGCs numbers. BITC treatment significantly alleviated RGCs damage in EAU mice, reducing neuronal apoptosis. Recent studies have suggested that activated microglia induce neuronal death in mice by mediating upregulation of TNF(He et al., 2025), indicating a close relationship between activated microglia and damage to retinal neurons. To further confirm whether the protective effect of BITC on neurons is achieved through environmental changes induced by microglia, we used an indirect co-culture system of BV2-primary RGCs to investigate the effect of the microenvironment after BV2 cell activation on primary RGCs, in order to minimize the interference caused by direct contact between drugs and primary RGCs. CM_LPS+IFN-γ_ from BV2 cells significantly exacerbated primary RGCs apoptosis. CM_BITC_ effectively mitigated this damage, markedly enhancing primary RGCs survival. However, direct cultivation of BITC with primary RGCs did not have a therapeutic effect. Therefore, we speculate that BITC inhibits primary RGCs impairment by regulating the secretion of BV2 cells. Previous studies have confirmed that pro-inflammatory cytokines secreted by activated microglia can directly induce neuronal injury and apoptosis (Chiu et al., 2018). Sivakumar et al. (2011) found that CM from microglia stimulated by hypoxia significantly enhanced the apoptosis of primary RGCs, whereas primary RGCs apoptosis was markedly reduced when this conditioned medium was treated with neutralizing antibodies against TNF-α/IL-1β(Sivakumar et al., 2011). This suggests that TNF-α and IL-1β secreted by BV2 cells are crucial factors in inducing primary RGCs apoptosis (Rana et al., 2026). Our results demonstrate that LPS + IFN-γ stimulation significantly increased the levels of pro-inflammatory factors TNF-α, IL-1β, and IL-6 in BV2 cells. BITC treatment led to a significant reduction in these inflammatory factor levels. Conversely, BITC treatment significantly upregulated the levels of BDNF and NGF in EAU mice and BV2 cells. As neurotrophic factors, BDNF and NGF initiate intracellular signaling cascades by activating specific tyrosine kinase receptors and the p75 neurotrophin receptor, thereby regulating neuronal survival, growth, differentiation, and synaptic plasticity (An L. et al., 2025). Research has shown that BDNF derived from microglia is essential for maintaining retinal homeostasis, and BDNF treatment significantly reduces neuronal degeneration caused by ethambutol (Cheng et al., 2025). In glaucoma studies, exogenous supplementation of BDNF markedly improved RGCs’ survival rates (Li et al., 2025). NGF is equally crucial for the function and visual maintenance of RGCs. Previous studies have shown that NGF can exert neuroprotective effects on microglia by acting on receptor tyrosine kinase 1 (TrkA) receptors (Latini et al., 2024). In addition, neurotrophic factors also have the ability to downregulate the expression of Major Histocompatibility Complex (MHC) II in microglia, which may help alleviate the toxic effects of microglial activation (Luo et al., 2022). Therefore, BITC may indirectly alleviate the degeneration of primary RGCs by reducing the levels of inflammatory factors in BV2 cells and promoting the secretion of neurotrophic factors in BV2 cells.

TREM-1 is a membrane receptor protein that exerts a pivotal amplifying function in immune and inflammatory responses, widely recognized as a key regulatory molecule in innate immunity (Dufrost et al., 2025). Recent studies indicate that TREM-1 is one of the key molecules connecting the immune system and neurodegenerative diseases. It not only modulates peripheral inflammation but also actively participates in neuroinflammatory processes within the central nervous system (Chen J. et al., 2025). In animal models of Parkinson’s disease, inhibition of TREM-1 reduced inflammatory cytokine secretion, alleviated dopaminergic neuronal damage, and improved motor function deficits (Song et al., 2025). We found that TREM-1 expression was significantly upregulated in the retina of EAU mice and BV2 cells stimulated with LPS and IFN-γ, suggesting that TREM-1 may play an important role in the progression of EAU. After BITC intervention, the expression of TREM-1 was inhibited. The PI3K/AKT signaling pathway is a key hub for regulating inflammatory responses (Khan et al., 2025). The application of PI3K inhibitors can alleviate its oxidative damage. This study found that BITC treatment significantly reduced the protein expression levels of p-PI3K and p-AKT in EAU mouse retinal tissue and LPS + IFN-γ stimulated BV2 cells, suggesting that BITC can effectively inhibit the activation of the PI3K/AKT pathway. Multiple studies have shown that the TREM-1 specific inhibitor LP17 significantly alleviates diseases such as cerebral hemorrhage and neuropathy, and inhibits excessive activation of the PI3K/AKT pathway. This indicates that Trem-1 may induce pathological changes in the central nervous system by mediating the activation of the PI3K/AKT pathway. This is consistent with our experimental results. After oe-TREM-1, the PI3K/AKT pathway was further activated, and in addition, the inhibitory effect of BITC was significantly weakened. Previous studies have explored the relationship between microglial phagocytosis and TREM-1. Lu et al. (2022) demonstrated that soluble TREM-1 stimulation significantly enhanced the phagocytic function of BV2 cells. It is crucial that this effect is mediated through the activation of the PI3K/AKT signaling pathway. PI3K inhibitors reversed the excessive activation and phagocytosis of BV2 cells induced by soluble TREM-1, thereby reducing neuronal damage. This indicates that the enhanced phagocytic effect of TREM-1 may be mediated through the PI3K/AKT pathway. Our results demonstrate that oe-TREM-1 further exacerbates oxidative stress, phagocytosis, and neuronal damage induced by LPS + IFN-γ, and partially reverses the antioxidant, anti-phagocytic, and neuronal damage effects of BITC, suggesting that BITC may exert therapeutic effects by regulating TREM-1.

This research has certain limitations. Firstly, BV2 cells *in vitro* experiments may not be able to fully replicate the phenotype and signaling characteristics of primary retinal microglia in EAU. Future research will use primary retinal microglia to further validate the mechanism of action of BITC, in order to more accurately reflect the true function of TREM-1/PI3K/AKT pathway in EAU neural injury in the retinal microenvironment. Secondly, this study used LPS + IFN-γ stimulation of BV2 cells to simulate the inflammatory activation of microglia in EAU. Although this model has been adopted by multiple studies in the EAU field, it cannot fully reproduce the complex immune network induced by T cells in EAU. Subsequent research will use stimulation conditions closer to the disease immune microenvironment for validation. In addition, although this study confirmed the functional relevance of TREM-1 in BITC protective effects through oe-TREM-1 experiments, the conclusion was not cross validated using TREM-1 loss of function experiments. Therefore, further functional validation is needed to determine the exact target effect of BITC on TREM-1. Finally, we must acknowledge that the research indicators of damage and apoptosis of retinal neurons in this study mainly reflect the level of neuronal structure and molecular level. These evidences cannot directly establish functional visual or retinal neuroprotection. Future research will improve electroretinogram and visual behavior testing to further evaluate the impact of BITC on visual function in EAU mice.

In summary, this study demonstrates that BITC mitigates breakdown of the BRB, oxidative stress, and neuronal damage in EAU. This protective effect is closely associated with the inhibition of the TREM-1/PI3K/AKT signaling pathway. Therefore, BITC, as a naturally occurring bioactive compound, may exert therapeutic potential by modulating the TREM-1/PI3K/AKT signaling pathway, offering a novel candidate molecule and research direction for EAU intervention.

## Data Availability

The original contributions presented in the study are included in the article/Supplementary Material, further inquiries can be directed to the corresponding author.
