# Supplementary material for: Benzyl isothiocyanate alleviates neural damage in experimental autoimmune uveitis by modulating the TREM-1/PI3K/AKT signaling pathway
Source: Front Pharmacol. 2026 Sep 24;17:1948992. doi: 10.3389/fphar.2026.1948992 (PMC13646128; doi:10.3389/fphar.2026.1948992)
Supplement: Supplementary file 1 [file Supplementaryfile1.docx]

Figure S1.（A-B）mRNA levels of BDNF and NGF.

Figure S2. Survival ability of primary RGCs after different CM treatments.

Figure S3. Negative control of secondary antibody staining only in mouse retinal sections (scale bar: 200 μm).
